# COVID-19, social determinants, and African American-White disparities: policy response and pathways forward

**DOI:** 10.1057/s41271-024-00528-8

**Published:** 2024-11-15

**Authors:** Lonnie R. Snowden, Genevieve Graaf

**Affiliations:** 1 School of Public Health, Health Policy and Management Division, University of California, Berkeley, CA USA; 2 School of Social Work, University of Texas at Arlington, Arlington, TX USA

**Keywords:** COVID-19, African Americans, Health policy, Economic policy

## Abstract

COVID-19 translated African Americans’ greater social, economic, and health-related risk, reflecting adverse Social Determinants of Health (SDOH), into greater COVID morbidity, hospitalization, and mortality, and it threatened to enlarge the very risks causing greater COVID suffering. However, following a federal policy response injecting trillions of dollars into the US economy, longstanding African American-White disparities in economic well-being, insurance coverage, vaccination rates, and evictions declined. On the other hand, troubling and consequential disparities in k-12 academic achievement and college attendance disparities widened. Continuous monitoring and careful research are needed to document and explain trajectories in social determinant disparities and to offer insight into how policy intervention can decrease continuing disparities in economic well-being, health care, and housing stability.

## Key messages:

- The COVID-19 pandemic did not enlarge most African American-White disparities in key indicators of well-being as had been widely and plausibly expected.
- A vigorous federal policy response, injecting trillions of dollars into established federal programs, avoided the expected shock for everyone and this largely prevented disparities from widening.
- Research is needed to document which programs had a beneficial impact on disparities and how their beneficial effects might be preserved or increased.

## Introduction

Throughout history, COVID-19 and other pandemics have spread most among disadvantaged populations [1] perpetuating and often increasing the very social and economic barriers leaving disadvantaged populations vulnerable at the outset. Socially and economically disadvantaged African Americans, through adverse Social Determinants of Health (SDOH), suffered especially from COVID-19. Among over 1 million deaths in the United States attributable to COVID-19, the largest death toll since the 1918–1920 flu pandemic [2]. As reported by the CDC in May of 2023, cumulative age-adjusted estimates show that Blacks’ death rate was 1.6 times greater than Whites’ and Blacks’ hospitalization rate was 2 times greater. African Americans’ COVID-19 illness rate was only 1.1 times greater than Whites despite hospitalization and death rate disparities [3], pointing to greater illness severity, less effective treatment, or both [4].

This greater COVID suffering was expected—as in the past—to translate into greater healthcare, social, and economic adversity [3]. Conceivably, however, as twenty first century vaccines and therapeutics combat illness, so might public policy mitigate healthcare barriers and social and economic social determinants of health (SDOH). How successful have modern policy measures been in mitigating SDOH-related disparities?

In what follows, we focus on African American populations which have suffered intergenerational racist exclusion [5]. African Americans do not receive non-COVID-19 vaccines and therapeutics as much as Whites [6–8] and it is reasonable to expect that the same would be true for COVID-19 vaccines and therapeutics. African American economic security [9], housing stability [10], and high school graduation rates [11] declined more than Whites’ during and after the Great Recession and it is reasonable to expect African Americans would suffer particularly from COVID-19’s non-biomedical sequelae. Yet a vigorous policy response might contain, or even reverse, the expected damage.

## COVID illness disparities and policy response

In response to the pandemic’s mounting toll, governmental authorities implemented policy interventions varying across state, regional, and local levels. In worldwide perspectives, policies [12] can be categorized in four groups as

- Containment and closure policies (school closures and movement restrictions)
- Economic policies (income support to citizens)
- Health system policies (emergency investments into healthcare and provisions to modify health care delivery)
- Vaccine policies (reducing cost of vaccination to individuals).

We focus here on federal policies targeting underlying social determinants which are critical to African Americans’ well-being. Social and economic disadvantages disproportionately experienced by African Americans are manifestations of cultural and institutional racism which offer enlightening insights for understanding COVID-19 health-related and social and economic challenges [13]. Here, we focus directly on COVID response policy as implemented and ask: How much did aggressive COVID-19 remediation and recovery policy prevent socioeconomic and health-related decline for everyone and, in this context, how much did it avoid exacerbating African Americans already great SDOH disadvantages?

Our approach is to identify key SDOH vulnerabilities from COVID where African American-White disparities are particularly problematic. Then, from a vast inventory of enacted COVID policies, we identify responses targeting these SDOH and ask, based on evidence available as this is written, how much they mitigated adversity for everyone and impacted disparities? We rely wherever possible on authoritative government sources charged with making official estimates (such as U.S. Department of Labor) and encourage readers to consult the sources as cited in the paper to learn how they were derived. To define social determinants, we turned to Healthy People 2030’s definition [14], which refers to conditions in the environments where people live, work, and age that affect their health, functioning, and quality of life. These conditions fall within five broad domains: Economic Stability, Education Access and Quality, Healthcare Care Access and Quality, Neighborhood and the Built Environment, and Social and Community Context [14]. We chose the most pandemic policy-relevant four as the focus of this review. Section headings presented below quote Healthy People’s SDOH Goals for each domain. Our timeframe extends from March 2020’s declaration of COVID-19 as a public health emergency through May of 2023 when the emergency ended.

Pandemic response policies were sprawling—the Coronavirus Aid, Relief, and Economic Security (CARES) Act, for example, injected billions into U.S. airlines to keep them flying through the pandemic. The hastily assembled policies were patchworks of components from established initiatives, repurposed and infused with new funds to contain a rapidly unfolding disaster by any means necessary [12]. We list major federal programs and provide links to informative summaries in Table 1, and we highlight pertinent features in text.

**Table 1:** Federal COVID-19 policy interventions

Policy	Key Provisions
The Coronavirus Aid, Relief, and Economic Security Act 6 March 2020	Key provisions are outlined in: National Conference of State Legislatures. COVID-19 Stimulus Bill: What It Means for States. Washington, D.C: April 2, 2020 [64]
The Families First Coronavirus Response Act 6 March 2020	Key provisions are outlined in: National Conference of State Legislatures. Summary of HR 6201–Families First Coronavirus Response Act. Washington, D.C: March 20, 2020 [65]
American Rescue Plan 11 March 2021	Key provisions are outlined in: National Conference of State Legislatures. “American Rescue Plan Act of 2021.” Resource. Washington, D.C., March 9, 2021 [66]

## Social determinant #1: “economic stability: help people earn steady incomes that allow them to meet their health needs”

As COVID-19 caused economic contraction from fear of contagion and from containment and closure policies aimed at mitigating infection spread. COVID-19 was forecast to bring about an economic crisis for everyone, African Americans especially [3]. African Americans’ already-higher unemployment rate spiked during previous recessions [15] and African Americans recovered more slowly from the 2007–2009 “Great Recession.” Ten years after the Great Recession Whites had recovered lost income [16] and lost wealth [9] but African Americans had not, and African Americans’ rates of forgone healthcare did not rebound as much as Whites’[17]. Data from early in the pandemic, March to September 2020, indicated that COVID-19 “has exacerbated some pre–COVID-19 economic and health disparities—including the wealth gap, housing disparity, lack of job security etc. among black Americans” [18].

The COVID-19 economic recovery proved for everyone quicker and stronger than expected—quicker and stronger than during the previous three non-COVID-19 downturns in 1990, 2001, and 2007—and African Americans benefited more than in the past [19]. African American unemployment declined more slowly than Whites during the pandemic but quickly returned to pre-COVID-19 levels and, in March 2023, hit a record low [20]. African Americans’ lower labor force participation, a longstanding concern [21], increased more than that of Whites according to the Council of Economic Advisors and African American workers showed marked wage gains from May 2021 to May 2022 [22].

Generous federal spending contributed to a better and more African American-friendly recovery than has occurred in the past. Remedial policies included increased unemployment assistance in the federal Coronavirus Aid, Relief, and Economic Security (CARES) Act, [23], a $2.2 trillion stimulus bill to blunt the economic damage set in motion by the global coronavirus pandemic, authorized direct payments of $1200 per adult plus $500 per child for individuals making up to $75,000, heads of households making up to $112,500, and couples filing jointly making up to $150,000. It was the first program of the COVID-19 era to place a moratorium on mortgage foreclosure and tenant evictions and it extended unemployment assistance and subsidized payroll for affected small businesses.

President Biden’s subsequent American Rescue Plan sent stimulus payments of $1,400, raised the Earned Income Tax Credit for working poor persons, and sent Child Tax Credit payments to millions of households. Helped by Supplemental Nutrition Assistance Program (SNAP) emergency allotments [24], these measures achieved documented reductions in children’s poverty rates population wide [25]. African Americans disproportionately benefited from these policies which provided individuals and families with sustaining financial resources and helped to avoid the downturn’s worst financial consequences as they helped to arrest a downward economic spiral [26].

According to the Council of Economic Advisors [22], the tightening labor market forced employers to attract workers partly though marked wage increases and these benefited Black workers especially. Although a significant wage gap persists, median wage growth for Black workers outpaced wage growth for Whites: For a one-year span preceding July 2022, median wage growth averaged 7.8% for Black workers and 6.3% overall. Additionally, Black workers moved up the ladder to traditionally higher-paying industries and occupations and African American self-employment rates in May 2022 were 15% above pre-pandemic levels.

African Americans suffered less than expected from the economic downturn caused by COVID-19 and sometimes even made small inroads in longstanding economic disparities. These gains might be short-lived—stalling or reversing—as interest rates rise to combat inflation and federal economic relief is rolled back. Indeed, amid low unemployment, African American unemployment rate in December 2023 was 1.6 times greater than the white rate [27] and reductions in child poverty, which were twice as great as those for Whites [25], are returning to pre-policy levels since expiration at the end of 2021 of the Child Tax Credit expansion [28]. Nevertheless, as the COVID-19 economic policies benefited everyone, they were associated also with African Americans’ better than expected performance.

## Social determinant #2: “health care access and quality: increase access to comprehensive, high quality healthcare services”

To increase healthcare access [29] and promote healthcare for COVID-19 and associated conditions, The American Rescue Plan expanded the Affordable Care Act. The Plan increased health insurance marketplace subsidies by capping premium contributions to 8.5% for nearly every income level, defraying health insurance costs for the unemployed, and providing new incentives for non-expansion states to accept expanded Medicaid. Authoritative projections indicate that 2.5 million uninsured people would receive coverage by 2023 and that premiums would decline for as many as 3.4 million low-income people [30].

Due to greater eligibility for incentives and strengthened outreach and enrollment efforts, the U. S. Department of Health and Human Services reported increased affordability for everyone in 2020 but especially for Blacks: 76% of African Americans without coverage could find a plan for less than $50 a month and 66% could find a plan with no monthly charge [31]. During a Marketplace Special Enrollment Period, African American enrollment increased from 9% in 2019 to 15% in 2021 [31] due to more generous subsidies and as states enlarged the pool of navigators to reduce administrative burdens and overcome distrust. States making the greatest gains—and where African Americans are overrepresented—had declined Medicaid expansion.

A public health emergency (PHE) provision under the Families Frist Coronavirus Response Act increased the federal share of Medicaid spending by 6.2% and required that Medicaid programs keep individuals continuously enrolled through the end of months in which the PHE was in effect [32], giving states enhanced federal funding for Medicaid beneficiaries and for payment of claims. Under this authority, Medicaid enrollment increased [33] and insurance rates dropped [34]. However, the PHE ended in May of 2023, ending federally enhanced Medicaid funding and requirements for Medicaid beneficiaries to re-enroll or re-apply for Medicaid coverage [33]. Disproportionately eligible for Medicaid, African Americans may have lost ground on coverage gains made under COVID-19 policies.

Low vaccination rates for non-COVID-19 diseases [35] are rooted in African Americans’ lack of trust in government and lack of access to the healthcare system [36], limiting prospects for successfully distributing the COVID-19 vaccines and therapeutics to African Americans to mitigate hospitalization and death from COVID-19 [37]. To overcome these obstacles critics recommend creative, culturally adapted, and vigorous educational campaigns to build trust in the health benefits of COVID-19 vaccines [37].

Extraordinary measures were indeed undertaken and appear to have increased African Americans’ belief that vaccines were necessary [38] and ultimately increased African Americans’ vaccine rates. According to the CDC data tracker [39], on November 26, 2022 among 75% of persons reporting race ethnicity, estimates are that about 88.2% of African Americans had received at least one dose of COVID-19 vaccine vs. 87.3% of Whites, and 84.9% of Blacks and Whites received one dose of a single-dose vaccine or a second dose in a two-dose series. Methodological barriers deny us complete confidence in estimation, but available data point to a remarkable achievement in closing initial disparities.

Several documented initiatives pointed to an aggressive outreach response. Community vaccination centers were centered in low-income communities, sometimes with mobile units to come as close as possible to community members. Retail pharmacies were enlisted through The Federal Retail Pharmacy Program [40] and they administered millions of doses—many in communities with established social vulnerability. Federally Qualified Health Centers (FQHCs) were made vaccine distribution sites and federal funds were made available to local communities to reach underserved populations.

Vaccination success did not transfer to receiving boosters. By the end of 2022, only 21.2% of Black adults versus 32.1% of Whites had received an updated (bivalent) booster dose, protecting against both the original virus and the Omicron variant BA.4 and BA.5 [41]. Disparities also remain in receipt of antiviral treatments, including Paxlovid—the most widely prescribed antiviral recommended for COVID-19-positive people with mild to moderate symptoms for persons at risk of developing severe illness. Between April and July 2022, CDC data show that the percentage of COVID-19 patients treated with Paxlovid was lower among Black individuals (21%) than among White (32%) [42].

Success was greater than expected for COVID-19 vaccinations as health system policies overcame reluctance. However, against a backdrop of limited overall success, earlier success did not transfer to ongoing vaccination and therapeutics. Lessons from vaccine success should be learned to determine policies and commitments necessary to overcome healthcare access barriers and mistrust in a high-stakes environment.

## Social determinant #3: “education access and quality goal: increase educational opportunities and help children and adolescents do well in school”

School closure policies prompted a shift to remote and hybrid instruction to avoid community spread of infection through students and teachers gathering in classrooms. The shift thwarted COVID-19 transmission to vulnerable teachers and vulnerable adults in children’s homes and community but sacrificed educational achievement as a cost. Standardized test pass rates in districts that were fully remote in 2020 or 2021 were 13 percentage points lower in mathematics and 8 percentage points lower in English Language Arts than districts that provided fully in-person instruction [43]. African American students were adversely affected especially; As measured by standardized tests, the disadvantage was greater in districts with more African American students [44]. Overall, educational achievement declined for African American students in particular and a longstanding achievement disparity between Black students and Whites widened [43]. Black students’ average growth reduction in math achievement was about 1.5 times greater than White students’ reduction [43].

Policy initiatives targeted losses in educational achievement and sought to address troubling disparities. The American Rescue Plan included more than $120 billion in funds to address pandemic learning loss and to invest particularly in districts and schools with students from underserved communities [45]. In upcoming years, research will need to assess the efficacy of the tutoring programs, summer learning initiatives, and other programs aimed at rebounding from school closures, emphasizing identifying intervention components pivotal to academic gains.

The pandemic also affected higher education institutions and students’ pursuit of postsecondary education and training. Spring enrollment of first-year Black students fell three times as much for Black as for White students between spring 2020 and spring 2021, and more than 4 times as much from Spring 2021 to 2022 [46]. The American Rescue plan’s Higher Education Emergency Relief Fund [47] prioritized funding to Historically Black Colleges, Minority-Serving Institutions, and community colleges and their students, providing emergency grants, discharging student debt or unpaid balances, and eliminating the withholding of transcripts for unpaid balances. And yet, first-year enrollment by Black students has continued to decline.

Longstanding disparities in educational achievement and college attendance were exacerbated by COVID-19. These disparities are especially troubling because they foretell widening disparities in key socioeconomic correlates: occupational success and income, incarceration, and poor health. Current initiatives should be monitored and evaluated, and new initiatives implemented to ensure that educational disparities do not widen and become an inflection point, erasing gains in other areas, and further diminishing African Americans prospects.

## Social determinant #4: “neighborhood and built environment: reduce the proportion of families that spend more than 30 percent of income on housing to ensure housing security”

Housing insecurity was perhaps policy makers’ greatest COVID built environment concern. Eviction was targeted because it increased the likelihood of exposure to COVID-19 and infecting others when evicted renters turn to friends and relatives for temporary housing—decreasing the ability to socially distance or self-quarantine and complicating rigorous hygiene measures needed to combat COVID [48]. Before COVID-19, African Americans lived in more crowded conditions [49], and African American renters were subject disproportionately to eviction filings and eviction judgment [50].

Although later it was struck down by the Supreme Court, the Centers for Disease Control and Prevention in 2020 issued an eviction moratorium for eight months, protecting nearly all renter households filing a declaration form. Many renters continued to be protected after expiration of the federal program by reinforcing state and local eviction moratoria for some or all of 2021 [51]. Eviction moratoria were associated with reduced COVID spread [52] and lifting them was associated with increased COVID spread [53].

Congressional action further helped renters through the Consolidated Appropriations Act of 2021 and the American Rescue Plan provided $46.5 billion in funding. One in seven low-income renters received rental assistance in 2021 and eviction moratoriums benefited African American renters especially [54]. Public policies enacted in response to the pandemic led to a sustained reduction in housing instability and were effective in helping those most in need. Although eviction policy at state levels was divided along racialized lines [55], eviction fell by as much as 50% from pre-Covid levels: they increased thereafter, slowly climbing to pre**-**COVID levels [56]**.** In the short run at least, aggressive federal, state, and local action prevented mass eviction and insured housing stability for millions of renters who are disproportionately African American**.**

## Confirming successful COVID policy response: recently published evidence

Recent publications relying on sources not cited here largely affirm the present findings and the picture they present. Using data from the Consumer Financial Protection Bureau’s Making Ends Meet Survey, Fulford [57] published the revealingly titled “The Pandemic Paradox: How the COVID Crisis Made Americans More Financially Secure.” Greater economic stability came about as “fewer Americans had difficulty paying the bills during the pandemic” (p. 135). Another volume again documents how the Child Tax Credit “reduced levels of poverty for all groups, it closed racial/ethnic gaps in poverty rates, and it led to consistently low poverty rates for all groups—at least from July to December, 2021”[58] (p.113).

Addressing education access and quality, Fulford [57] again documented learning loss from school closure during the pandemic, especially among Black students. Fulford too was pessimistic that the American Rescue Plan’s 120 billion dollar investment will redress lost academic achievement, seeing little prospect especially that vulnerable students will overcome their learning losses. Fulford [57] documents a sharp decline in evictions during the pandemic, attributing it initially to administrative closure of eviction processing procedures but later to eviction-restricting policy initiatives.

Regarding healthcare access and quality, Kaiser Family Foundation researchers [59] confirmed that continuous Medicaid enrollment and other measures raised insurance coverage rates for everyone and closed African American-white disparities. The Foundation continues to monitor rising uninsurance rates from continuous enrollment’s expiration. Except for improving education access and success, evidence continues to mount for COVID-19 policies success for everyone and sometimes especially for African Americans.

## COVID policy response acted directly on social determinants of health to produce beneficial change

COVID policies followed existing pathways of action in targeting specific social determinants as a sense of urgency dictated repurposing of existing programs. This allowed bypassing the many stages of designing, vetting, and negotiating legislation required to win approval of new initiatives. African American benefit was often a built-in feature because African Americans lower incomes and higher poverty rates entitle them disproportionately to receive benefits.

Along with direct cash payments, existing programs, including extended unemployment insurance, greater Earned Income Tax Credit payments, the Child Tax Credit and SNAP eligibility expansion, funneled resources directly to African American and other recipients. The differential impact of income support was felt immediately in bank balances, as generous federal spending boosted African Americans’ checking accounts more than Whites’ [60]. Federal support cushioned the impact of African Americans’ lower incomes and lesser wealth [3].

Medicaid adjustments and marketplace subsidy adjustments, as well as Federally Qualified Health Centers support, increased health care access by increasing eligibility and generosity of benefits. Enlisting the Federal Retail Pharmacy Program and deploying community vaccination centers to boost vaccination rates and implementing eviction moratoriums demonstrate how, beyond established antipoverty programs, overlooked providers and sources of vulnerability, can be recruited to reduce racial disparities. Resourceful use of existing programs offers an expeditious pathway to meaningful, targeted improvement for African Americans and other vulnerable populations.

## Implications for post-COVID SDOH and disparity policy

COVID-19 policy responses should be carefully studied as natural experiments, confirming as rigorously as possible which policy instruments do improve well-being and decrease key disparities in economic well-being, health care, and housing stability. Variation in state policy response, as well as the dismantling over time of COVID-related PHE measures, allows for quasi-experimental research addressing causality within “treatment–control” and “treatment-reversal” frameworks. Rigorous investigation can establish the impacts of emergency and social welfare policies on well-being and disparities.

Once confirmed, favorable COVID policy responses will prove that key SDOH outcomes and disparities, sometimes considered intractable, are responsive to within-reach policy initiatives. As the child tax credit [61] significantly reduced child poverty, so COVID-19 policy response might point to levers which can successfully address key SDOH outcomes and disparities. Economic stimulus policies can improve labor market outcomes and reduce earnings disparities. These and other important gains should be weighed along with others against a predominant concern, inflation risk, stimulating the economy. Aggressive measures for increasing the availability of health insurance coverage and making healthcare services more accessible bring more people needed care and occasion measurable reductions in key disparities. Eviction can be greatly reduced with disproportionate benefit to African Americans, reducing the many vulnerabilities flowing from unstable housing. When results are confirmed, advocates should press for these policy measures which bring tangible benefit to the lives of African Americans and others.

## Conclusions

The COVID-19 pandemic struck the US population forcefully, but its impact was mitigated by a forthright policy response. Vigorous government response was not inevitable: critics strongly objected to curtailment of personal freedom from containment and closure policies, mask mandates, and vaccine policies [62]. Some demanded austerity from fear of rising deficits from lost revenue [63], but proponents of generous spending prevailed. Everyone withstood economic and social adversity better than expected and, amid a favorable response overall, African-White disparities did not widen and sometimes even shrank. Economic disparities did not increase as in past recessions where African American unemployment rates particularly spiked. Indeed, African Americans entered the labor market in greater numbers, saw increases in earnings which persisted after allowing for inflation, and made other modest gains. Insurance coverage and initial vaccination disparities did not widen as expected and even declined, although therapeutics did display customary patterns of racial disparity. Eviction disparities, primed to surge, fell instead. These responses demonstrate that targeted economic and health system policies can make inroads in disparities even when rooted in long term, pervasive, and deeply entrenched structural disparities. We must continue to monitor efforts to reverse this trend lest educational achievement disparities define COVID’s legacy—a Black Swan event with potentially detrimental intergenerational consequences. However, on balance, a large scale COVID-19 social and economic policy response not only prevented a continuing social and economic catastrophe from following in COVID’s wake, it created selected improvements in frustratingly enduring conditions of adversity. Through enlightened response, COVID’s legacy can be more than mass illness and death and include policy lessons for bettering the lives of many Americans and African Americans especially.
